# Ultrasound-Assisted Through-Mask Electrochemical Machining of Hole Arrays in ODS Superalloy

**DOI:** 10.3390/ma13245780

**Published:** 2020-12-17

**Authors:** Guoqian Wang, Yan Zhang, Hansong Li, Jian Tang

**Affiliations:** 1 School of Mechanical and Power Engineering, Nanjing Tech University, Nanjing 211816, China; wanggq@njtech.edu.cn (G.W.); tangjian_1126@163.com (J.T.); 2 College of Mechanical and Electrical Engineering, Nanjing University of Aeronautics and Astronautics, Nanjing 210016, China; hsli@nuaa.edu.cn

**Keywords:** ultrasound-assisted through-mask ECM, cavitation, hole array, ODS superalloy

## Abstract

Micro-hole arrays have found wide applications in aerospace, precision instruments, and biomedicine. Among various methods of their production, including mechanical, laser, and electrical discharge, electrochemical machining (ECM) is considered the most lucrative due to its wide processing range, high surface quality, and excellent productivity. In particular, ultrasound-assisted through-mask ECM exhibits an enhanced machining precision due to ultrasonic cavitation, which promotes the removal of the electrolytic products and bubbles. In this study, the equation of cavitation bubble oscillation was derived and numerically solved to study the influence of six different parameters on the ultrasonic cavitation and electrolysis process, and their optimal values were determined. The feasibility of the proposed ultrasound-assisted through-mask ECM technology with the optimized parameters was experimentally corroborated by the fabrication of a high-quality hole array in an oxide dispersion strengthened (ODS) MA956 superalloy.

## 1. Introduction

Hole arrays are becoming increasingly used in state-of-the-art applications of aerospace, precision instruments, and biomedicine. In particular, hole arrays are adopted in the thin-walled components of aero-engines made of high-strength and high-temperature materials. The performance of such components can be severely deteriorated by array-machining defects, such as the heat-affected zone, cracks, and plastic flow [1,2].

Hole arrays can be produced by conventional machining, laser machining, electrical discharge machining, and electrochemical machining (ECM). Conventional machining is extensively used due to its high-cost efficiency, practicality, and stability, but its productivity/machining efficiency needs to be further improved for the large-scale production of hole arrays [3]. Laser machining exhibits a higher controllability and applies to the micro-machining of different complex shapes; however, there is the problem of the recast layer after machining [4]. Electrical discharge machining has the benefits of low stress, no burring, and the processability of high-hardness materials [5]. However, there are still some specific drawbacks and problems, such as electrode wear, the recast layer, and the exchange of dielectrics, which have to be resolved [6]. ECM occupies an essential position in micro-hole array processing due to its high machining performance. ECM ensures a higher stability of the machining process without the heat-affected zone generation, machining stress, and the limitation of material hardness [7,8]. As an ECM technology, through-mask ECM (TMECM) is primarily intended for micro-hole array processing with restraining the machinable region by using a thin insulation sheet with a specific pattern, and TMECM has been widely used to produce micro-structured components such as micro-dimples, hole arrays and micro-grooves. The research focuses of TMECM include the analysis of the forming process, the optimization of mask characteristics, and the improvement of electrolyte exchange [9]. In an analysis of hole-forming process, Li et al. [10,11] established a numerical model and optimized the process parameters of TMECM, and then high-quality hole arrays were successfully fabricated on a structure of titanium alloy and molybdenum materials. To further improve mask performance, Kunar et al. [12] reported a new reusable masked tool to fabricate a micro square pattern by ECM and obtained ideal results. On the other hand, Mahata et al. [13] proposed a novel process for micro-dimple arrays by TMECM with low-aspect ratio masks that could promote the quality of micro-dimple arrays and save expenditure for a thick mask. Though TMECM has been successfully used in micro-hole array processing, some serious problems such as the consistent precision of large-scale machining and stray-current corrosion remain unsolved.

In particular, the application of TMECM to micro-hole array fabrication in an oxide dispersion strengthened (ODS) superalloy named MA956, which is used in gas-turbine combustion chambers has the issue that an insoluble floccule formed by electrolysis may adhere to the workpiece, which impedes reaction proceeding. Furthermore, the cathode does not move during TMECM, and a long machining time inevitably increases the machining gap and cause severe stray-current corrosion. Ultrasound has stirring and cavitation effects, and it can promote the transport of an electrolyte solution and the elimination of the electrolysis products. These features make the ultrasound method lucrative for improving the efficiency and precision of ECM. Pa [14] carried out an in-depth study on ultrasonic electrochemical micro-finishing and developed an electrochemical micro-finishing technology using composite ultrasound/magnetic field. The designed test bed was also experimentally validated. Bhattacharyya et al. [15] developed a microtool vibration system for electrochemical micromachining and studied the influence of vibration frequency, pressure amplitude, and the concentration of the electrolyte solution on the precision of micro-hole processing. To enhance ECM, Patel et al. [16] combined the pulsed current and ultrasonic to drill deep holes in 6061-T6 aluminum. This technique enhanced part quality by reducing the surface roughness parameter (Ra) from 2.5 to 1 μm and the taper angle of the drilled holes from 11° to 1°. In addition, Zhu et al. [17] applied ultrasonic vibration to electrochemical drill-grinding and machined micro-holes with a surface roughness of 0.31 μm and a taper of less than 0.6°. All above investigations confirmed the beneficial effect of ultrasonics on ECM. Therefore, it is necessary to use ultrasonics in TMECM. Wang et al. [18] promoted mass transfer in TMECM using ultrasonic stirring, and a well-defined micro-pits array with 30 μm on a large scale was demonstrated with the above-discussed method.

Despite the above research progress, ultrasound-assisted TMECM is still at the exploratory stage given its structural uniqueness. Based on the literature review, we first analyzed the working principle of ultrasound processing, assessed the ultrasound parameters by simulation, and discussed the influence of electrolyte parameters on ultrasonic cavitation. Next, an experimental device for ultrasound-assisted TMECM was designed and validated. Finally, a high-quality hole array structure was fabricated on an ODS MA956 superalloy using the above procedures.

## 2. Numerical Analysis of Ultrasound-Assisted TMECM

### 2.1. Principles of Ultrasound-Assisted TMECM

**Figure 1:**
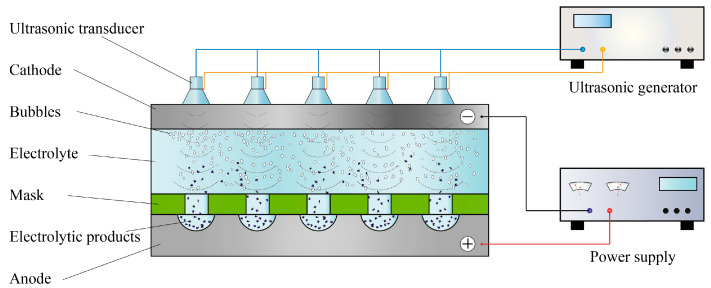
Working principle of ultrasound-assisted through-mask electrochemical machining (TMECM).

An ultrasound-assisted TMECM system consists of a cathode, an anode, a mask, an electrolyte flow channel, and an ultrasonic transducer, as shown in Figure 1. A mask with a specific hole array pattern tightly clings to the surface of the workpiece through mechanical force on the epoxy resin sheet during processing, and the electrolyte solution rapidly flows between the mask and cathode, carrying away the processing by-products and cooling them. The electrolytic reaction occurs in the exposed portion of the workpiece in the electrolyte solution. The ultrasonic transducers are put on the cathode to generate ultrasonic waves, and then the ultrasonic cavitation works on promoting the removal of electrolytic products and bubbles.

### 2.2. Principle of Ultrasonic Cavitation

**Figure 2:**
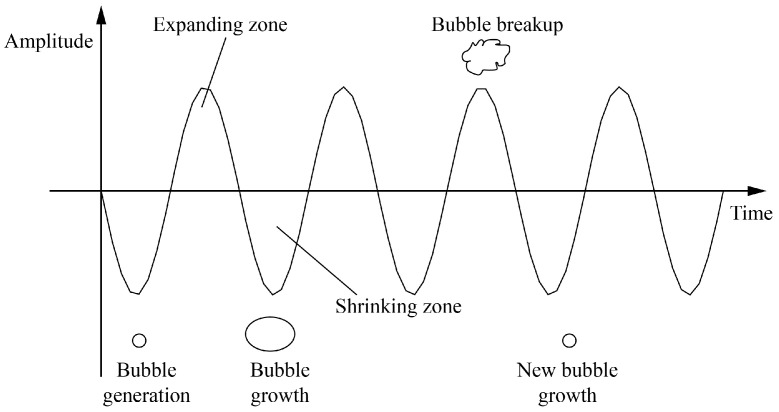
Schematic of cavitation mechanism.

Cavitation is considered the primary mechanism of interaction between ultrasonic waves and a medium, which consists of two processes: (i) the generation and unique motion of cavitation bubbles and (ii) subsequent bubble collapse under ultrasonic action (Figure 2). Let the sound pressure *P* of ultrasonic wave imposed on the electrolyte solution be defined as *P* = *P_f_*·sin(2*πft*), where *P_f_* and *f* are the ultrasonic pressure amplitude and frequency, respectively, while *t* is time. Assume that the initial pressure on the electrolyte (*P*_0_) varies within the range from *P*_0_ − *P_f_* to *P*_0_ + *P_f_*. The negative half-cycle from 0 to *P*_0_ − *P_f_* implies an increasingly negative pressure on the electrolyte solution. If *P_f_* is sufficiently large, the intermolecular distance can increase to infinity, thus creating cavitation bubbles. When the intensity of pressure is *P*_0_ − *P_f_*, bubbles grow to their largest size. After that, the bubbles are compressed by the continually increasing positive pressure, and the degree of compression is the highest at *P*_0_ + *P_f_*. This periodic oscillation causes high-speed bubble collapse, with temperature and pressure jumps that result in respective mechanical and thermal effects [19,20].

Ultrasonic cavitation can be instrumental for TMECM for the following reasons. Firstly, it promotes the removal of the electrolysis products and the cleaning of the electrode surface, thus facilitating the electrolysis process. Secondly, it causes the collapse of cavitation bubbles already present or newly generated in the electrolyte solution, thus increasing the electrode surface area. Thirdly, ultrasonic cavitation is conducive to the constant motion of a solution, reducing the concentration polarization and increasing the current density and efficiency.

To elucidate the influence of the ultrasound and ECM system parameters on cavitation, we elaborated a dynamic model of ultrasonic cavitation for TMECM. Suppose that the air and water vapor in the bubbles are ideal gases, while the liquid is incompressible. Disregarding the gravity action, the bubble wall oscillation can be treated as a spherically symmetric one. The equation of cavitation bubble motion under the adiabatic conditions is derived from the principle of conservation of energy and Rayleigh equation for the bubble growth is as follows [21,22,23]:

(1) $$R(\frac{d^{2}R}{dt^{2}})+\frac{3}{2}{\left(\frac{dR}{dt}\right)}^{2}=\frac{1}{\rho }[(P_{0}+\frac{2\sigma }{R_{0}}-P_{v}){\left(\frac{R_{0}}{R}\right)}^{3k}+P_{v}+P-P_{0}-\frac{2\sigma }{R}-\frac{4\mu }{R}\cdot \frac{dR}{dt}]$$

Considering the bubble expansion damping, the above formula is reduced to:

(2) $$\begin{array}{r} R(\frac{d^{2}R}{dt^{2}})+\frac{3}{2}{\left(\frac{dR}{dt}\right)}^{2}=\frac{1}{\rho }[(P_{0}+\frac{2\sigma }{R_{0}}-P_{v}){\left(\frac{R_{0}}{R}\right)}^{3k}+P_{v}+P-P_{0}-\frac{2\sigma }{R}-\frac{4\mu }{R}\cdot \frac{dR}{dt}]\\ +\frac{R}{\rho c}\cdot \frac{d}{dt}[(P_{0}+\frac{2\sigma }{R}){\left(\frac{R_{0}}{R}\right)}^{3k}-P] \end{array}$$

Equation (2) describes bubble wall oscillation under the action of ultrasonic waves, with the initial conditions *t* = 0, *R* = *R*_0_, and *dR/dt* = 0. Here, *R* and *R*_0_ are the instantaneous and initial radii of the cavitation bubble, respectively; *P* is the sound pressure; *P*_0_ is the electrolyte pressure; *P_v_* is the vapor pressure within the bubble; *c* is the sound velocity in the liquid; *σ* is the surface tension coefficient of the electrolyte; *μ* is the liquid viscosity coefficient; *ρ* is the density of the electrolyte; *k* is the adiabatic index; and $\frac{4\mu }{\rho R}$ is the term of viscous loss.

In accordance with Equation (2), the temperature and pressure of the electrolyte liquid affect both the ultrasonic cavitation and electrolysis reaction processes. Therefore, the optimization of these parameters is necessary for ultrasound-assisted TMECM. As a realistic electrolysis medium, a 10% NaCl electrolyte solution was investigated, and four parameters—namely temperature, pressure, viscosity coefficient, and surface tension—were varied, as were two parameters of the ultrasound waves (frequency and pressure amplitude), with their further optimization for the ultrasound-assisted TMECM.

Equation (2) is a second-order nonlinear ordinary differential equation that has no analytic solution. To study the influence of each parameter on the cavitation bubble wall oscillation, it was solved iteratively using the MATLAB software (R2017a) and plotted as *R*(*t*)/*R*_0_ vs. *t* curves, where *R*(*t*)/*R*_0_ is the ratio of instantaneous and initial radii of the cavitation bubble, respectively, which characterizes the intensity of bubble growth/contraction and the intensity of ultrasonic cavitation, while *t* is the time of one cycle of ultrasonic cavitation.

#### 2.2.1. Influence of Ultrasound Parameters

**Figure 3:**
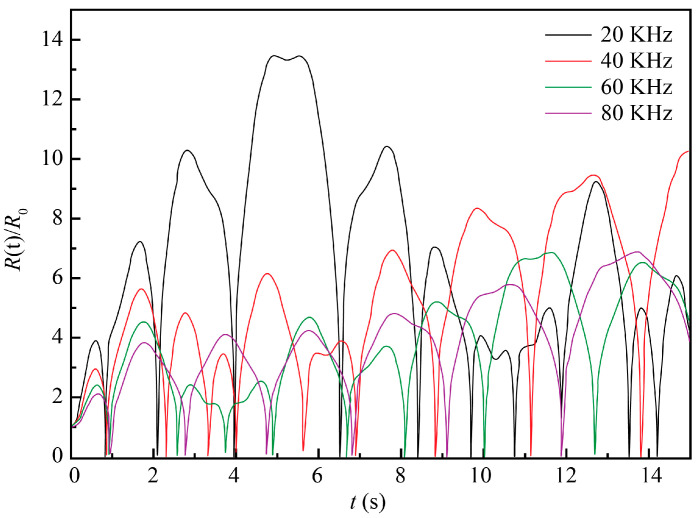
Evolution of cavitation bubble oscillation amplitude at four different frequencies and constant ultrasound pressure amplitude (*P_f_*) = 0.2 MPa.

**Figure 4:**
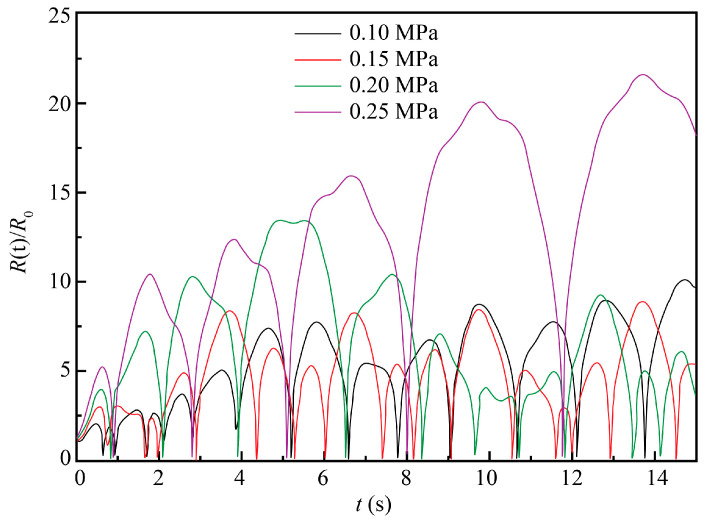
Evolution of cavitation bubble oscillation amplitude at four different ultrasound pressures and constant ultrasound frequency (*f*) = 20 kHz.

The 10% NaCl solution at the standard atmospheric pressure and room temperature of 20 °C was taken as the ECM system, with the initial conditions *t* = 0, *R* = *R*_0_, and *dR/dt* = 0. Other parameters were as follows: *ρ* = 1071 kg/m^3^, *σ* = 0.076 N/m, *R*_0_ = 50 μm, *P*_v_ = 2.34 × 10^−3^ Pa, *μ* = 1.153 × 10^−3^ Pa∙s, *k* = 1.33, and *P*_0_ = 1.013 × 10^5^ Pa. The numerical simulation was performed for the following values of two independent variables: ultrasound frequency (*f*) values of 20, 40, 60, and 80 kHz and ultrasound pressure amplitude (*P_f_*) values of 0.1, 0.15, 0.2, and 0.25 MPa. The obtained results are plotted in Figure 3 and Figure 4, respectively.

As seen in Figure 3, an increase in the ultrasound frequency resulted in the gradual reduction of the bubble oscillation amplitude, i.e., the relative ratio of its instantaneous and initial radii *R*(t)/*R*_0_. The highest value of the latter was observed at the ultrasound frequency of 20 kHz, which implied that smaller ultrasound frequencies were more lucrative for cavitation. Additionally, as the ultrasound frequency increased, the time needed for the bubble to achieve the largest diameter also increased. With other conditions being constant, the variation of bubble radius decreased and the ultrasonic cavitation was weakened with the ultrasound frequency. Moreover, higher ultrasonic frequencies led to the higher energy consumption of the ultrasonic wave propagation in the liquid. Therefore, lower ultrasound frequencies are preferred for ultrasound-assisted TMECM.

As seen in Figure 4, as the ultrasound power/pressure increased, the variation of bubble radius also increased with an enhancement of cavitation. Therefore, to maximize ultrasonic cavitation, higher values of the ultrasound power/pressure are preferred for ECM. However, if the ultrasonic energy is too high, bubbles will become too large in the expansion zone, which may result in their immediate collapse in the contraction zone, thus impairing the ultrasonic cavitation process. Given this, intermediary values of ultrasound pressure in the range from 0.15 to 0.2 MPa seem to be the most instrumental for the steady and efficient ECM process.

#### 2.2.2. Influence of Pressure of Electrolyte Solution

The pressure of the electrolyte (*P*_0_) solution controls ultrasonic cavitation by influencing the transient pressure of a bubble. In this study, during ultrasound-assisted TMECM, the pressure at the liquid outlet of the centrifugal pump (*P_a_*) was measured using pressure gauges. Meanwhile, the instantaneous electrolyte pressure (*P*_0_) at any point in the processing (i.e., ultrasonic cavitation) zone was assessed via the liquid outlet pressure using the Bernoulli equation:

(3) $$P_{0}=P_{a}+\frac{\rho }{2}(v_{2}^{2}-v_{1}^{2})+\rho g(h_{2}-h_{1})$$

where *v*_2_ is the flow rate at the liquid outlet, *v*_1_ is the flow velocity at any point in the ultrasonic cavitation zone, and *h*_2_ − *h*_1_ is the height difference.

**Figure 5:**
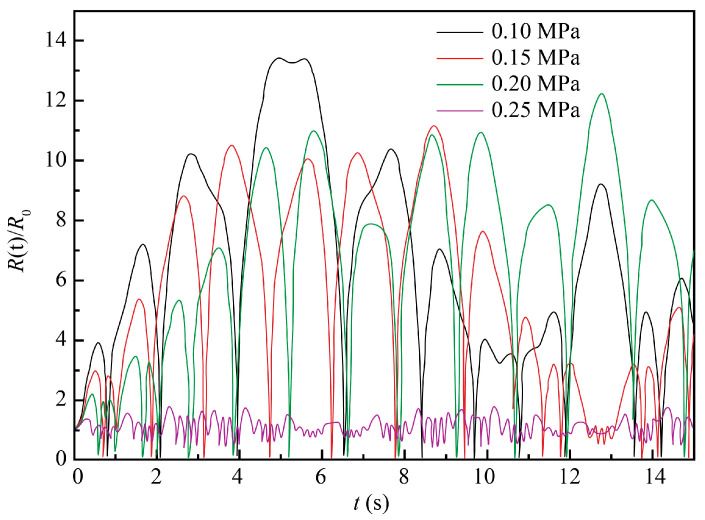
Evolution of cavitation bubble oscillation amplitude at four different pressure values of the electrolyte solution.

Similar to the earlier simulation conditions, the 10% NaCl solution at the standard atmospheric pressure and room temperature of 20 °C was taken as the ECM system, with the initial conditions *t* = 0, *R* = *R*_0_, and *dR/dt* = 0. Other parameters were as follows: *ρ* = 1071 kg/m^3^, *σ* = 0.076 N/m, *R*_0_ = 50 μm, *P_v_* = 2.34 × 10^−3^ Pa, *μ* = 1.153 × 10^−3^ Pa s, *k* = 1.33, *f* = 20 kHz, and *P_f_* = 0.2 MPa. A numerical simulation was performed for different values of electrolyte pressure (*P*_0_), namely 0.1, 0.15, 0.2, and 0.25 MPa. The obtained results are plotted in Figure 5.

As seen in Figure 5, as the electrolyte pressure increased, the intensity of bubble oscillation decreased and the cavitation process faded. When the electrolyte pressure was larger than the sound pressure level, the cavitation became significantly weakened or could hardly occur, which corroborated the present findings. In practice, an electrolyte solution pressure should be chosen based on comprehensive considerations. Too-low values may enhance the ultrasonic cavitation process but fail to remove the electrolysis by-products and dissipate the generated Joule heat, which is also unfavorable for achieving a good machining effect. Though cavitation was found to weaken as the electrolyte pressure increased, the curves in Figure 5 corresponding to 0.15 and 0.20 MPa differ only slightly. That is, changes of instantaneous static pressure within the intermediate range had no significant impact on the ultrasonic cavitation. Therefore, an electrolyte solution pressure level should be selected in a such way that the instantaneous static pressure in the processing zone does not exceed the sound pressure of the ultrasonic wave. On the other hand, it should be sufficient to carry away the electrolysis by-products and the Joule heat without weakening the ultrasonic cavitation process.

#### 2.2.3. Influence of Viscosity and Surface Tension Coefficients

The 10% NaCl solution at the standard atmospheric pressure and 20 °C was taken as the ECM system, with the initial conditions *t* = 0, *R*(t) = *R*_0_, and *dR/dt* = 0. Other parameters were as follows: *ρ* = 1071 kg/m^3^, *σ* = 0.076 N/m, *R*_0_ = 50 μm, *P_v_* = 2.34 × 10^−3^ Pa, *k* = 1.33 and *P*_0_ = 1.013 × 10^5^ Pa, *f* = 20 kHz, and *P_f_* = 0.2 MPa. The numerical simulation was performed for three values of the viscosity coefficient *μ*, namely 1.0 × 10^−3^, 2.0 × 10^−3^, and 3.0 × 10^−3^ Pa∙s and three values of the surface tension coefficient *σ* (0.076, 0.1, and 0.15 N/m).

**Figure 6:**
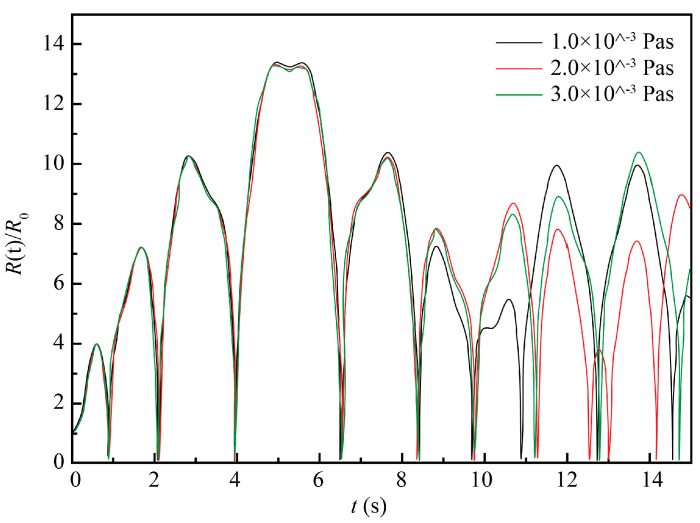
Evolution of cavitation bubble oscillation amplitude for three different values of the viscosity coefficient and constant surface tension coefficient of the electrolyte (*σ*) = 0.076 N/m.

**Figure 7:**
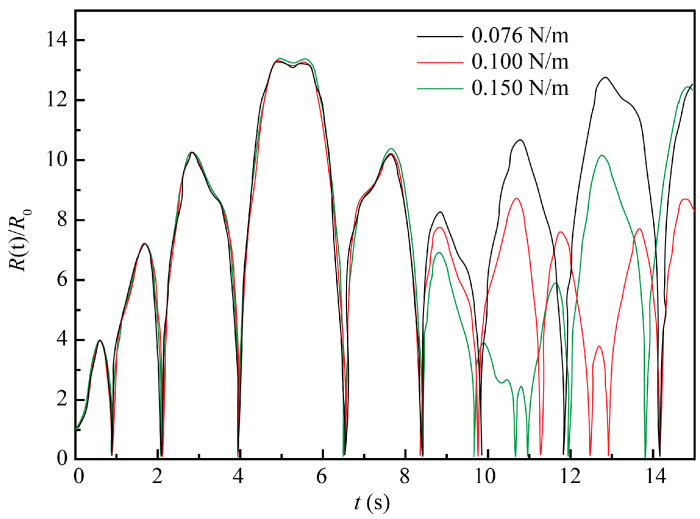
Evolution of cavitation bubble oscillation amplitudes for three different values of the surface tension coefficients and constant the liquid viscosity coefficient (*μ*) = 1.153 × 10^−3^ Pa∙s.

As shown in Figure 6 and Figure 7, liquid surface tension and viscosity have a slight impact on the amplitude of bubble oscillation. Under general experimental conditions, the influence of these two factors on ultrasonic cavitation is neglected. For the 10% NaCl solution used in this study, there were little changes in the liquid surface tension and viscosity under normal machining conditions. Therefore, the influence of these two factors was neglected in the further analyses.

#### 2.2.4. Influence of the ECM System Temperature

Many aspects should be considered when studying the impact of the temperatures on ultrasound-assisted ECM because such a process covers both an electrolysis reaction and machining performance. Therefore, it is expedient to experimentally assess the temperature effect patterns for specific materials. Additionally, temperature affects ultrasonic cavitation by influencing the viscosity coefficient of the electrolyte, surface tension, and vapor pressure in the cavitation bubbles. To further analyze the effect of the temperature on the ultrasonic cavitation, the viscosity coefficients of a 10% NaCl solution, surface tension, and vapor pressure in the bubble were calculated for the temperatures of 20, 30, 50, and 60 °C (see Table 1).

**Figure 8:**
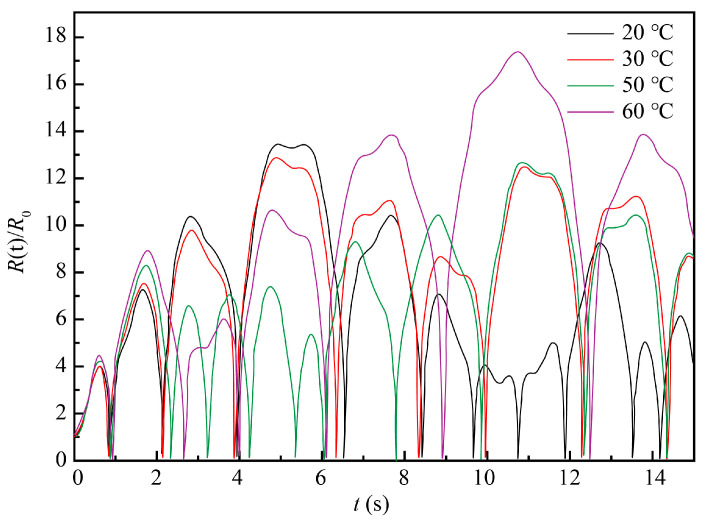
Evolution of cavitation bubble oscillation amplitude at four different temperatures.

The 10% NaCl solution at the standard atmospheric pressure and 20 °C was taken as the ECM system, with the initial conditions *t* = 0, *R*(t) = *R*_0_, and *dR/dt* = 0. Other parameters were *ρ* = 1071 kg/m^3^, *σ* = 0.076 N/m, *R*_0_ = 50 μm, *P_v_* = 2.34 × 10^−3^ Pa, *μ* = 1.153 × 10^−3^ Pa∙s, *k* = 1.33, *P_v_* = 1.013 × 10^5^ Pa, *f* = 20 kHz, and *P* = 0.2 MPa. The numerical simulation was performed for the following temperatures: 30, 40, 50, and 60 °C. The obtained results are plotted in Figure 8.

As shown in Figure 8, the amplitude of cavitation bubble oscillation and the cavitation effect were found to have complex relationships with temperature. It was difficult to find a clear trend or optimal value just based on the above-shown results. While lower temperatures were less favorable for the electrolysis reaction, ECM requires an electrolyte solution being heated to a certain temperature that should not be too high either. Therefore, more experimental research was conducted to ensure the optimal temperature of ECM.

## 3. Experimental

### 3.1. The Anode Material

In this study, the anode material where the hole array was produced by ultrasound-assisted TMECM was the ODS MA956 superalloy (Luhao Group Co., Ltd., Shanghai, China). It is widely used in gas-turbine combustion chambers, advanced energy-conversion systems, and other applications that require rigorous conditions due to its exceptional strength and resistance to corrosion, carburization, and oxidation at temperatures over 1100 °C. The production of this alloy involves the high-energy milling of metal powders and strengthening by yttrium oxide dispersoid. Its composition (in wt.%) is as follows: 74% Fe, 20% Cr, 4.5% Al, 0.5% Ti, and 0.5% yttrium oxide Y_2_O_3_. The mechanical properties are: a tensile strength (annealed) of 650 MPa, a yield stress (annealed) of 550 MPa, and an elongation at break of 9%.

### 3.2. Experimental Setup

**Figure 9:**
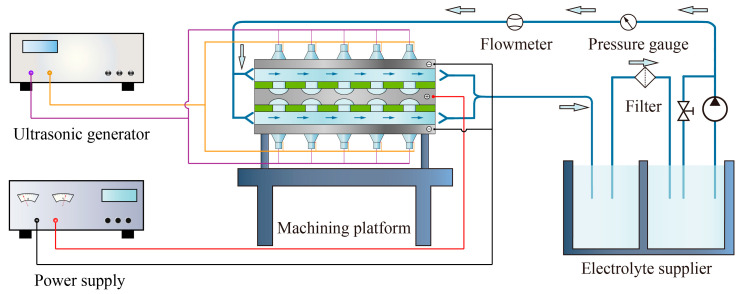
Experimental setup.

Based on the above analysis and schematic depicted in Figure 1, an original experimental setup was developed for TMECM of the ODS MA956 superalloy, as shown in Figure 9. The experimental setup consisted of an electrolyte circulation system, a high-power pulse power supply (Yisheng Electronics Co., Ltd., Shanghai, China), ultrasound equipment, a machining platform, and a clamping device. More detailed information is given in Figure 9. To reduce the conicity of the machining hole and increase the machining efficiency, a symmetrical double-sided processing mode was usually applied. The ultrasound equipment (Jiayuanda Tech. Co., Ltd., Shenzhen, China) included several ultrasound transducers and an ultrasonic generator that generated frequency and power-adjustable ultrasonic waves with a maximum power of 3 kW. The machining platform and clamping device ensured the safe and reliable occurrence of the electrolysis reaction. The cathode material was steel, the anode (workpiece) material was the MA956 superalloy, and the thickness of the workpiece was 0.5 mm. The mask was an epoxy resin sheet with the hole radii of 2 mm. The 10% NaCl solution was taken as the electrolyte solution and was used in the machining platform with an electrolyte circulation system.

## 4. Results and Discussion

### 4.1. Experiment Investigation on Parameters

**Figure 10:**
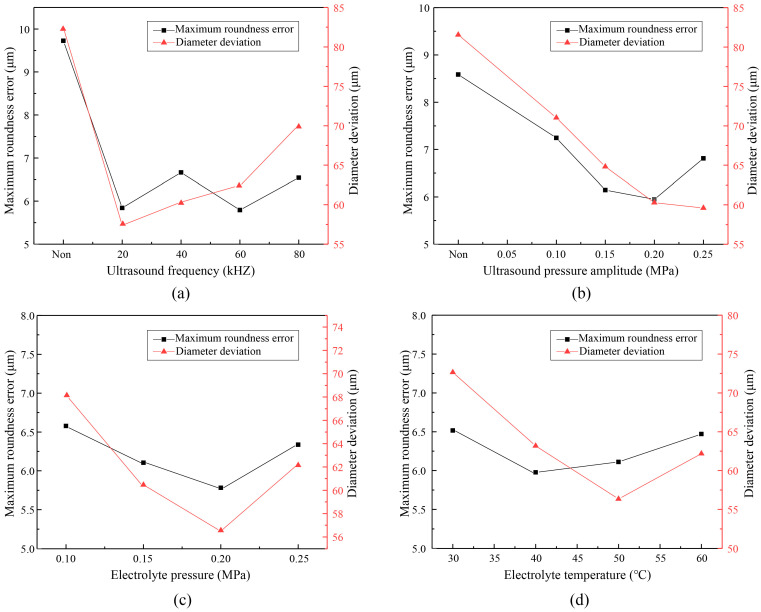
Experimental results with different parameters. (**a**) Diameter deviation and roundness error with ultrasound frequency; (**b**) Diameter deviation and roundness error with ultrasound pressure amplitude; (**c**) Diameter deviation and roundness error with electrolyte pressure; (**d**) Diameter deviation and roundness error with electrolyte temperature.

According to the previous numerical analysis, there were four parameters—namely ultrasonic frequency, ultrasonic pressure amplitude, electrolyte pressure and temperature—that had significant effects on cavitation. All the parameters needed further experimental verification, especially the optimal electrolyte temperature, which had not been confirmed. Therefore, experiment investigations of ultrasound-assisted TMECM were implemented with different parameters, while the diameter deviation and maximum roundness error of fabricated holes were measured to evaluate the parameters. The experimental results are shown in Figure 10.

As shown in Figure 10, the experimental results basically agreed with the numerical analysis conclusions. When the cavitation effect was strengthened, the through-mask ECM effect was better. Of course, what was not similar was that each parameter affected the diameter deviation and roundness error. Figure 10a indicates that ultrasound could improve the machining results, and the increase of ultrasonic frequency had little effect on the roundness error but aggravated the diameter deviation. The increase of ultrasonic pressure was beneficial for the decrease of diameter deviation and roundness error, as shown in Figure 10b, but the roundness error began to rise when the ultrasonic pressure reached 0.25 MPa. As shown in Figure 10c, the influence trend of electrolyte pressure on the diameter deviation and roundness error was similar, and the optimum results appeared at 0.2 MPa. From Figure 10d, it can be seen that the diameter deviation declined gradually with the increase of temperature but the machining quality would be deteriorated if the temperature exceeded 50 °C. Given all this information, a set of optimized parameters can be given: an ultrasonic frequency of 20 kHz, an ultrasonic pressure of 0.2 MPa, an electrolyte pressure of 0.2 MPa, and an electrolyte temperature of 50 °C. The detailed values of all parameters adopted for Figure 10 are listed in Table 2.

### 4.2. Experiment Investigation on Hole Array Structure

A hole array structure was fabricated by ultrasound-assisted through-mask ECM with the optimized parameters. The mask had a 6 × 8 hole array structure with a hole radii of 2 mm. The 10% NaCl solution was taken as the electrolyte, with an ECM treatment time of 3.5 min. The ultrasound transducers were oscillators with a rated frequency/power of 20 kHz/1 kW and a sound pressure level of about 0.2 MPa. Based on the above findings, the remaining parameters of the ultrasound waves and electrolyte solution are listed in Table 3.

**Figure 11:**
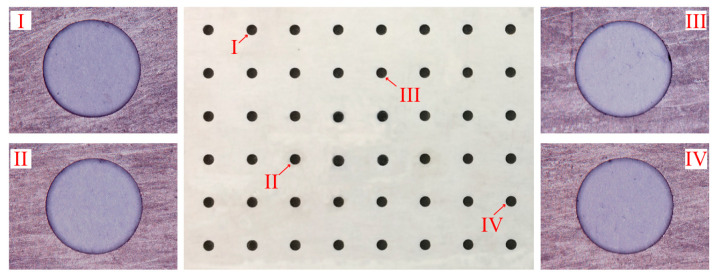
Hole array structure fabricated by TMECM.

The obtained experimental results, based on the fabrication of a 6 × 8 hole array in the MA956, are illustrated in Figure 11. Each hole produced by ECM was shown to have a regular round shape, a clear boundary, and a slight conicity. Magnified views of some holes and cross-sectional views of their side walls were captured using a three-dimensional profilometer (DVM5000, Leica, Heidelberg, Germany). The respective results are depicted in Figure 11.

**Figure 12:**
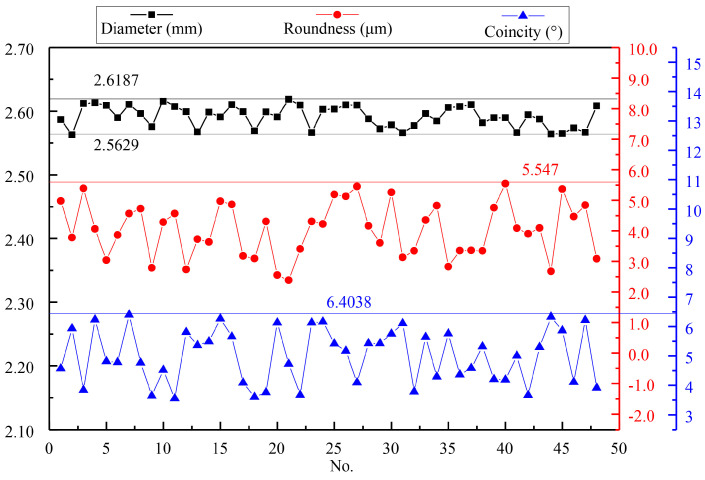
The measured results of the hole array structure.

For the quantitative evaluation of the hole array’s machining quality, the measured dimensions, roundness deviations, and conicity values of each hole are shown in Figure 11. As seen in the Figure 12, the limit deviation of diameters was 0.0558 mm, the roundness error did not exceed 6 μm, and the maximum conicity was only 6.4038°. These results indicated a high machining quality.

## 5. Conclusions

Micro-hole arrays have found wide applications in aerospace, precision instruments, and biomedicine. Ultrasound-assisted through-mask ECM is considered a great prospective method of array machining due to its wide processing range, high surface quality, and excellent productivity. In this study, the influence of six different parameters on the ultrasonic cavitation and electrolysis process was analyzed numerically according to the equation of cavitation bubble oscillation. The analyzed results indicated that the ultrasound frequency, ultrasound pressure, electrolyte solution pressure, and electrolyte solution temperature had significant effects on ultrasonic cavitation, but liquid surface tension and viscosity had slight impacts on the cavitation. In the meantime, the optimal value of each parameter was determined. Then, ultrasound-assisted through-mask ECM technology with optimized parameters was experimentally corroborated. A high-quality hole array (6 × 8) was fabricated in an ODS MA956 superalloy. The limit deviation of diameters was 0.0558 mm, the roundness error did not exceed 6 μm, and the maximum conicity was only 6.4038°. The experimental results demonstrated the feasibility of the proposed ultrasound-assisted TMECM setup and the optimization of its parameters.

## Figures and Tables

**Table 1:** Values of the viscosity coefficient, surface tension, and vapor pressure at different temperatures of a 10% NaCl electrolyte solution.

Temperature (°C)	Viscosity Coefficient (×10^−3^ Pa∙s)	Surface Tension (N/m)	Vapor Pressure (Pa)
20	1.153	0.0760	2.34 × 10^3^
30	0.950	0.0745	4.25 × 10^3^
50	0.670	0.0715	1.23 × 10^4^
60	0.570	0.0700	1.99 × 10^4^

**Table 2:** The values of all parameters adopted for Figure 10.

Measurement Index	Ultrasonic Frequency (kHz)					Electrolyte Pressure (MPa)			
	Non	20	40	60	80	0.10	0.15	0.20	0.25
Maximum roundness error (μm)	9.726	5.844	6.663	5.793	6.542	6.578	6.104	5.781	6.337
Diameter deviation (μm)	82.4	57.3	60.3	62.5	70.1	68.3	60.5	56.6	62.2
Measurement index	Ultrasonic pressure amplitude (MPa)					Electrolyte temperature (°C)			
	Non	0.10	0.15	0.20	0.25	30	40	50	60
Maximum roundness error (μm)	8.584	7.245	6.144	5.951	6.811	6.064	5.976	6.110	6.471
Diameter deviation (μm)	81.5	71.0	64.8	60.3	59.6	72.6	63.2	56.4	62.3

**Table 3:** Optimized ECM treatment parameters.

Parameter	Value
The anode metal	MA956
Voltage, V	25
Electric current efficiency, %	100
Frequency of pulse power supply, Hz	400
Duty ratio of pulse power supply, %	20
Temperature of the electrolyte, °C	50
Pressure of the electrolyte, MPa	0.2
